# Direct Dengue Virus Genome Sequencing from Antigen NS1 Rapid Diagnostic Tests: A Proof-of-Concept with the Standard Q Dengue Duo Assay

**DOI:** 10.3390/v15112167

**Published:** 2023-10-28

**Authors:** Francisco-Javier Pérez-Rodríguez, Florian Laubscher, Valentin Chudzinski, Laurent Kaiser, Samuel Cordey

**Affiliations:** 1 Laboratory of Virology, Department of Diagnostics, Geneva University Hospitals & Faculty of Medicine, University of Geneva, 1205 Geneva, Switzerland; francisco.perezrodriguez@hcuge.ch (F.-J.P.-R.); florian.laubscher@hcuge.ch (F.L.); valentin.chudzinski@hcuge.ch (V.C.); laurent.kaiser@hcuge.ch (L.K.); 2 Swiss Reference Centre for Emerging Viral Diseases, Geneva University Hospitals, 1205 Geneva, Switzerland; 3 Division of Infectious Diseases, Geneva University Hospitals, 1205 Geneva, Switzerland; 4 Geneva Centre for Emerging Viral Diseases, Geneva University Hospitals, 1205 Geneva, Switzerland

**Keywords:** dengue virus, antigen rapid diagnostic tests, sequencing

## Abstract

With nearly half of the world’s population being at risk of infection, dengue virus represents a major global health issue. The use of dengue antigen rapid diagnostic tests (Ag-RDTs) represents an alternative to PCR methods for the diagnosis of acute infections since they display excellent sensitivities and specificities and can be performed outside the laboratory. The high genetic diversity of the dengue virus genome represents a challenge for vaccine development, and the progressive expansion of this virus into previously nonendemic regions justifies the implementation of a genomic surveillance program. In this proof-of-concept study, we show the feasibility of sequencing dengue virus genomes directly from positive Ag-RDT (Standard Q Dengue Duo Test assay, *n* = 7) cassettes stored up to 31 days at room temperature after testing. For 5 of the 7 samples, a high number of reads were obtained allowing phylogenetic analyses to be carried out to determine not only the serotypes (dengue 1, 2, 3 and 4 were detected) but also the genotypes. Furthermore, in one sample, our unbiased metagenomic next-generation sequencing approach made it possible to detect epizootic hemorrhagic disease virus sequences, an arthropod-transmitted virus in ruminants. To conclude, as such an approach requires no cold storage or freezing of samples, dengue Ag-RDTs represent a very pragmatic and robust alternative for the genomic surveillance of dengue virus.

## 1. Introduction

Dengue virus are enveloped, positive-sense single-stranded RNA viruses belonging to the *Flaviviridae* family and divided into four serotypes (dengue 1, 2, 3 and 4) based on antigenic differences. This pathogen represents a global threat since the prevalence of dengue infections has increased significantly in recent years, with almost half of the world’s population (Sub-Saharan Africa, Asia, Latin America) being at risk of infection and an estimated ~100 million symptomatic cases each year [1,2]. This progression will likely continue in parallel with the constant expansion of the habitat of its vector *Aedes* spp. mosquitoes into previously nonendemic areas.

The diagnosis of dengue disease is usually performed either via PCR or serology (i.e., IgM and IgG testing) depending on the onset of symptoms. Although such methods are implemented in most routine laboratories, both require specific and expensive equipment, and a turnaround time of several hours, and they are not well suited to field investigations in certain regions. This is why, in addition to these classical approaches, different companies have developed dengue fever rapid diagnostic tests (RDTs), making it possible to carry out a diagnosis in a few minutes (the test is able to be performed outside of the laboratory) thanks to the rapid detection of dengue antigen (Ag-RDT), IgM and/or IgG.

The dengue virus genome shows high genetic diversity [3], both within and between the four different serotypes, which represents a major challenge for vaccine development. Therefore, there is a need for a global dengue genomic surveillance program to rapidly identify new emerging lineages. Furthermore, one or more dengue serotypes are frequently dominant in some dengue-endemic areas. Therefore, if in such regions the dengue serotyping is based solely on PCR screening using specific sets of primers and probes, it would not be possible to identify the introduction of new genotypes which could possibly belong to the same locally dominant serotype but are sufficiently antigenically divergent to result in an outbreak (i.e., homotypic dengue virus reinfections [4]) and have a significant local clinical impact. Currently, sequencing protocols are based on whole blood, plasma, or sera, requiring adequate storage and transport conditions of clinical samples, which in certain situations can represent a serious limitation to sequencing.

Our laboratory uses the Standard Q Dengue Duo Test assay (SD Biosensor, Haryana, India) in case of urgent diagnostic requests from clinicians. In a recent comparative study of six dengue RDTs (108 specimen tested, mainly dengue 2 serotype), this assay showed the highest antigen (NS1) and IgM sensitivities (87.0% and 84.3%, respectively; combined NS1 + IgM = 99.1%), the highest negative predictive value (NS1 = 68.2%, IgM = 63.8%, combined NS1 + IgM = 96.8%) and a positive predictive value > 98% [5]. Furthermore, the authors examined dengue NS1 specificity and showed that this assay did not cross react with Zika and chikungunya virus supernatant, which is in line with the manufacturer’s specifications for dengue NS1 (absence of cross reactivity with Zika virus, chikungunya virus, yellow fever virus (vaccine) and Japanese encephalitis virus; specificity = 98.7%). As the feasibility of sequencing directly from Ag-RDTs has recently been demonstrated for SARS-CoV-2 and considered as a potential gamechanger of SARS-CoV-2 genomic surveillance [6,7], the aim of this study was to assess the possibility of performing sequencing directly from dengue-positive Ag-RDTs tested in our routine laboratory during our 2023 summer period. To reflect the reality in the field as closely as possible, all positive Ag-RDTs were considered for sequencing, independently of the viral load obtained subsequently via real-time RT-PCR (Table 1). Furthermore, RDTs were stored for up to 4 weeks at room temperature to evaluate the possibility of shipment from the field.

## 2. Materials and Methods

### 2.1. Samples

Sera were collected from patients returning from travel between June and August 2023 and sent to our routine laboratory (Geneva University Hospitals, Geneva, Switzerland) at the request of physicians for dengue Ag-RDT and IgM/IgG-RDT analysis. Sera were collected between 3 and 8 days post-symptom onset. All dengue-positive Ag-RDT cassettes (*n* = 7) during this period were include in this proof-of-concept study. After RDT analysis, sera were stored at −20 °C.

### 2.2. Dengue NS1 Antigen RDTs

Dengue Ag- and IgM/IgG-RDTs were performed using the Standard Q Dengue Duo Test assay (two devices) (SD Biosensor, Haryana, India) with 100 µL and 10 µL of sera, respectively, according to the manufacturers’ instructions. Positive Ag-RDTs were then stored at room temperature up to 31 days before sequencing (range: 7–31 days, Table 1).

### 2.3. Unbiased Metagenomic Next-Generation Sequencing (mNGS) from RDTs Strips and Sequence Analysis

**Figure 1:**
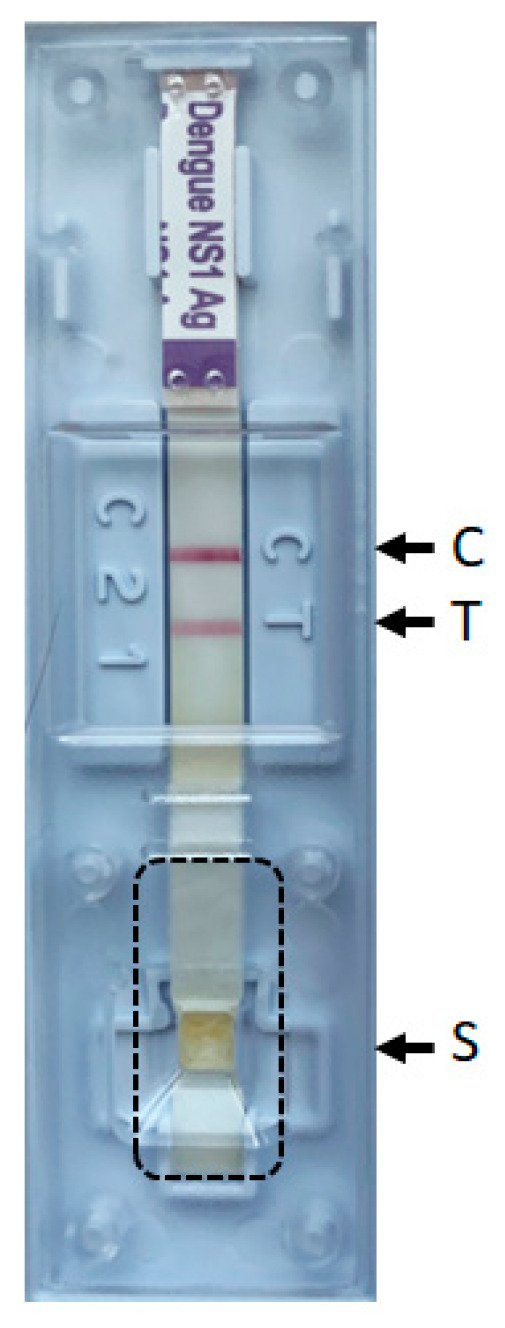
Standard Q Dengue antigen assay. Dotted lines show the excised part of the strip used for direct sequencing. S: sample well, C: control line, T: NS1 line.

The cover of the Ag-RDT cassette was removed. Then, the part of the strip corresponding to the sample well (i.e., where the sample is added and absorbed on the sample pad) was excised using a scalpel (Figure 1), placed in 250 µL of PBS and incubated for 10 min at room temperature (vortexed regularly). After a brief spin down, 200 μL of supernatant was recovered and spiked with 20 µL of standardized canine distemper viruses (CDV, internal mNGS process control). RNA virus genome extraction was performed with TRIzol according to the manufacturer’s instructions (Invitrogen, Carlsbad, CA, USA). The RNA pellet was resuspended in 10 µL of RNase-free water (Promega, Dubendorf, Switzerland). Then, libraries were prepared using the TruSeq total RNA preparation protocol (Illumina, San Diego, CA, USA). Library concentrations were measured using Qubit (Life Technologies, Carlsbad, CA, USA), and the size distribution of fragments was controlled using a 2200 TapeStation (Agilent, Santa Clara, CA, USA). Then, libraries were loaded on the MiSeq platform (Illumina) using the 2 × 75 bp paired-end protocol. Reads were analyzed using a bioinformatics pipeline designed to detect all vertebrate viruses (based on the Virosaurus database [8] and a de novo assembly sequencing approach, as previously described [9]. The maximum likelihood method and the Tamura-Nei model [10] were used to construct the phylogenetic tree based on the complete genome for dengue 1, 2 and 3 serotypes (Figure S1). The analyses were conducted in MEGA X [11].

The dengue virus genome sequence detected from sample 2 was deposited in GenBank under the accession number OR448787.

### 2.4. Real-Time RT–PCR

The viral genomes were extracted from 190 µL of sera using the NucliSENS easyMAG (bioMérieux, Geneva, Switzerland) spiked with 10 µL of standardized CDV, as previously described [12]. Nucleic acids were recovered in an elution volume 25 μL. The dengue real-time RT-PCR analysis (using the dengue specific primers DENV-F, DENV-R1, and DENV-R2 and probe P from the Trioplex Real-time RT-PCR Assay, designed by the Centers for Disease Control and Prevention [13] was performed with 10 µL of eluate using the Superscript^®^ III Platinum^®^ One-Step qRT-PCR Kit (Invitrogen) in a QuantStudio 5 instrument (Applied Biosystems, Rotkreuz, Switzerland) under the following cycling conditions: 50 °C for 30 min, 95 °C for 2 min, 45 cycles of 15 s at 95 °C and 1 min at 60 °C.

## 3. Results

Using our sequencing protocol directly from the Ag-RDT we successfully obtained dengue virus specific sequences for six of the seven positive Ag-RDTs analyzed, even after the Ag-RDT cassettes remained at room temperature for more than 4 weeks. RDT (antigen and IgM/IgG), unbiased mNGS and PCR results are summarized in Table 1. The sequencing analysis allowed us to determine for six of the seven samples the dengue virus serotype (dengue 1, 2, 3 and 4 serotypes were detected). In addition, for five samples, a high number of reads were obtained allowing phylogenetic analyses to be carried out to further determine the genotypes based on the complete genome (Figure S1). Of note, the de novo analysis did not provide more information than those obtained with our bioinformatics pipeline designed to detect all vertebrate viruses. Interestingly, our unbiased mNGS analysis further reported the presence of epizootic hemorrhagic disease virus (468 reads, 55.05% genome coverage, all segments detected)- and human pegivirus (9 reads, 6.85% genome coverage)-specific reads in samples 4 and 6, respectively. 

Retrospective real-time RT-PCR analyzes were positive for six of the seven samples (Table 1), the negative one corresponding to the one for which no dengue virus-specific reads were detected via unbiased mNGS. The number of dengue-specific reads obtained only partially reflects the Ct values reported, a well-known phenomenon in unbiased mNGS investigations (a series of variables, both specific to a given sample and not, are known to impact the mNGS analytical sensitivity [14]). Remarkably, dengue virus genotypes could be obtained by unbiased mNGS for samples with Ct values slightly above 31 (Table 1).

## 4. Discussion

This proof-of-concept study proves the feasibility of sequencing the dengue virus genome directly from the Standard Q Ag-RDT. Although only a small number of samples were available for this study, a high number of reads were obtained for five of the seven samples allowing phylogenetic analyses to be carried out to determine not only the serotypes but also the genotypes. Here we used a benchmarked unbiased sequencing approach used by our group to perform many virome investigations [9,15,16,17], but other approaches such as amplicon-based sequencing protocols (i.e., targeted sequencing) could be used to obtain high depth and genome coverage. Further studies including a large panel of dengue virus RDTs with suitable sensitivity and specificity values to justify their use in the field are needed. Indeed, users may decide to use other NS1 Ag-RDTs. However, an important point to consider is to ensure that the latter are highly specific to the dengue virus NS1 Ag and do not cross react with, for example, other flaviviruses circulating in the same geographical region. Interestingly, our unbiased mNGS analysis further reported in one case (sample 4) the presence of epizootic hemorrhagic disease virus-specific reads, an arthropod-transmitted virus responsible for the epizootic hemorrhagic disease in domestic and wild ruminants [18]. This highlights the added value of using an unbiased mNGS approach, which also allows the detection/surveillance in parallel of other viral agents (i.e., co-infections). It is important to mention that the fact of being able to carry out direct sequencing from dengue NS1 RDTs may certainly facilitate molecular epidemiology surveillance programs but has no direct impact on the management or outcome of the source patient.

In conclusion, (1) our protocol requires no cold storage or freezing of samples and (2) we successfully obtained dengue virus sequences even after positive Ag-RDT cassettes were stored for more than 4 weeks at room temperature (i.e., the viral genomic material is preserved in the Ag-RDTs), which gives enough time to send positive Ag-RDTs from the field to a partner laboratory with sequencing capacities; thus, this approach represents a very pragmatic alternative for the genomic surveillance of dengue virus.

## Figures and Tables

**Table 1:** Summary of the RDTs, mNGS and real-time RT-PCR results. For mNGS results, the total number of reads, percentage of human reads, number of reads mapping to dengue virus genome, % of genome coverage and depth reported are those obtained with our bioinformatics pipeline based on the Virosaurus database.

Sample	Traveler Returning From	RDT (Standard Q Duo) Results			Unbiased mNGS										Real-Time RT-PCR Ct Values
		Antigen NS1 (Number of Days between Onset of Symptoms and Sample Collection)	IgM	IgG	Number of Days the Ag-RDT Remained at Room Temperature before Being Processed for Sequencing	Total Number of Reads	Human Reads (%)	Number of Reads Mapping to Dengue Virus Genome	Genome Coverage (%)	Depth	Serotype	Genotype	Best Matched GenBank Reference (Accession Number)	Ability to Perform Phylogenetic Analysis (yes/no)	
1	Thailand	positive (3)	negative	negative	31	17,565,488	11.112	1616	88.9	10	dengue 3	G III	MW788884	yes	11.3
2	Singapore	positive (5)	positive	positive	28	22,705,906	7.115	2860	99.4	19	dengue 2	Cosmopolitan	MW512481	yes	20.3
3	Cuba	positive (5)	positive	negative	23	25,572,466	8.082	8	5.1	1	dengue 4	undetermined	OR162320	no	21.9
4	Mexico	positive (unknown)	equivocal	negative	8	25,912,062	5.918	70	17.48	2	dengue 1	G V	GQ868510	yes	31.3
5	Costa Rica	positive (6)	equivocal	negative	15	7,569,600	7.086	409	54.87	4	dengue 1	G V	OQ603263	yes	21.5
6	Cambodia/Indonesia	positive (8)	positive	negative	14	7,179,842	6.517	0	0	0	undetermined	undetermined	not applicable	no	undetected
7	Cambodia/Indonesia	positive (8)	equivocal	negative	7	33,997,742	6.03	72	16.59	2	dengue 1	G I	MZ619038	yes	31.2

## Data Availability

The raw sequence data were deposited in the NCBI Sequence Read Archive under BioProject accession number PRJNA1005611.

## Supplementary Materials

The following supporting information can be downloaded at: https://www.mdpi.com/article/10.3390/v15112167/s1, Figure S1: Phylogenetic tree of dengue virus serotypes 1 (**a**), 2 (**b**) and 3 (**c**) complete genome sequences. Samples collected in this study are in red color. GenBank accession numbers are provided in brackets.
