# Supplementary figures and images for: Direct Dengue Virus Genome Sequencing from Antigen NS1 Rapid Diagnostic Tests: A Proof-of-Concept with the Standard Q Dengue Duo Assay
Source: Viruses. 2023 Oct 28;15(11):2167. doi: 10.3390/v15112167 (PMC10674465; doi:10.3390/v15112167)

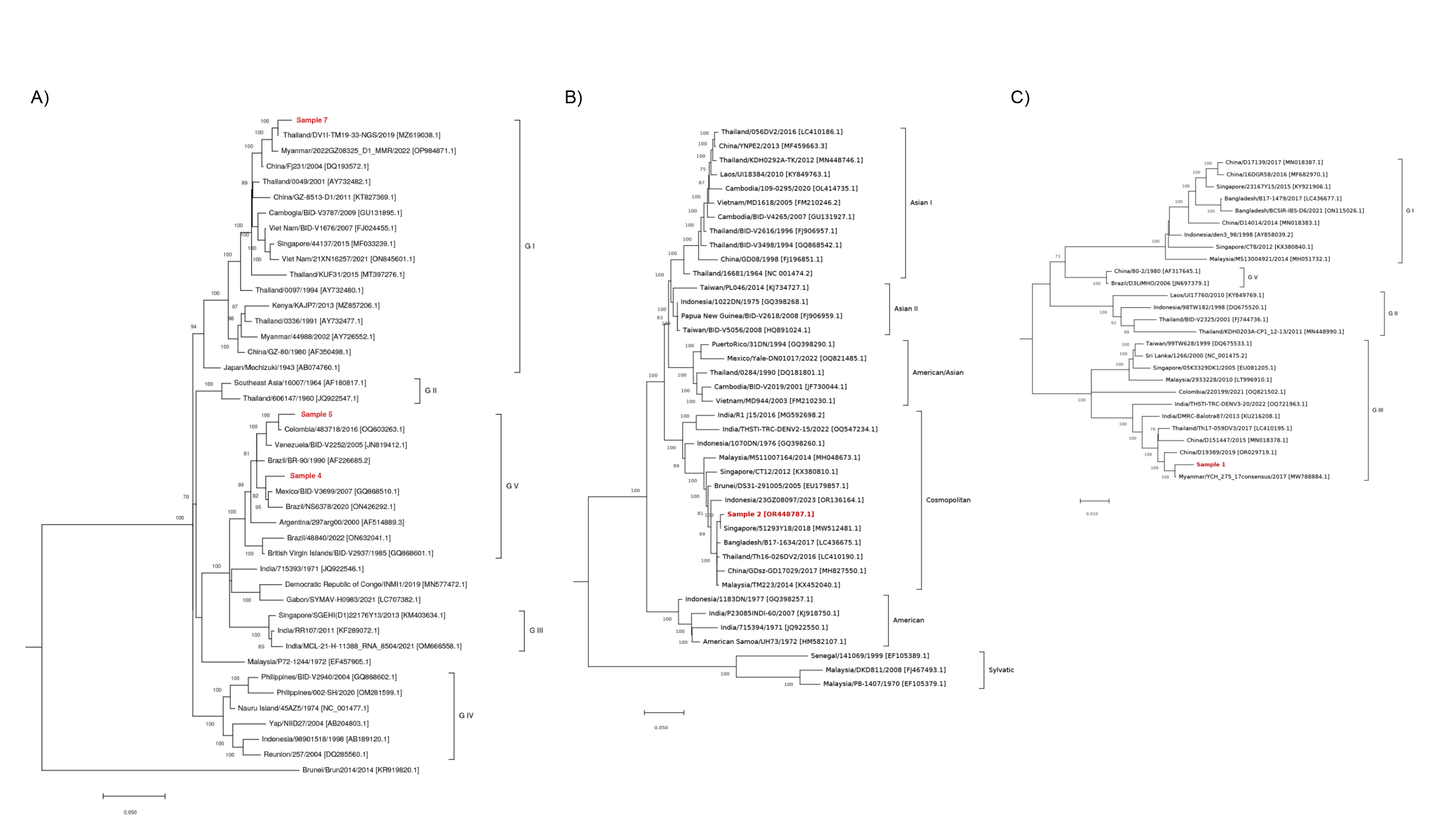

Supplement: Supplementary file 1 [file viruses-15-02167-s001.zip › viruses-2659156-supplementary.tif]
